# KCNJ2 Facilitates Clear Cell Renal Cell Carcinoma Progression and Glucose Metabolism

**DOI:** 10.1155/ijog/2210652

**Published:** 2025-04-24

**Authors:** Qiyue Zhao, Zhengshu Wei, Guanglin Yang, Liwei Wei, Hao Chen, Zelin Cui, Naikai Liao, Min Qin, Jiwen Cheng

**Affiliations:** ^1^Department of Urology, First Affiliated Hospital of Guangxi Medical University, Nanning, Guangxi Zhuang Autonomous Region, China; ^2^Department of Surgery, Guangxi Medical University, Nanning, Guangxi Zhuang Autonomous Region, China; ^3^Department of Urology, Liuzhou Workers' Hospital, Guangxi Medical University, Liuzhou, Guangxi, China

**Keywords:** clear cell renal cell carcinoma, glucose metabolism, KCNJ2, progression

## Abstract

**Background:** Clear cell renal cell carcinoma (ccRCC) is marked by aggressive characteristics and a poor prognosis. The involvement of KCNJ2, an inward rectifying potassium channel, in the progression of ccRCC, along with its potential roles in immune modulation and metabolic pathways, remains unclear.

**Methods:** The Cancer Genome Atlas (TCGA) database was utilized to analyze the gene expression, clinicopathological characteristics, and clinical relevance of KCNJ2. The prognostic value of KCNJ2 in ccRCC was evaluated with Kaplan–Meier survival analysis and receiver operating characteristic curve analyses. The TCGA-KIRC dataset was utilized to analyze tumor microenvironment (TME), focusing on tumor-infiltrating immune cells and immunomodulators. The biological functions of KCNJ2 were investigated in vitro using CCK-8, flow cytometry, wound healing, transwell, qRT-PCR, and Western blotting assays.

**Results:** KCNJ2 expression was notably higher in ccRCC than in normal kidney tissues, with increased levels associated with advanced tumor stages. However, KCNJ2 did not exhibit obvious prognostic value. Coexpression analysis identified associations with genes implicated in energy metabolism. Analysis of the TME and immune profile indicated a link between KCNJ2 expression and immune cell infiltration, along with particular immune checkpoints. *In vitro* studies demonstrated that KCNJ2 overexpression enhanced cell proliferation, migration, invasion, glucose production, and ATP generation.

**Conclusion:** KCNJ2 plays a crucial role in ccRCC progression through affecting glucose metabolism and immune responses. Our findings reveal the functional role of KCNJ2 in promoting tumor progression and metabolic reprogramming in ccRCC, highlighting its therapeutic potential as a novel target for ccRCC treatment. Further studies are essential to clarify the mechanisms by which KCNJ2 affects ccRCC biology and to evaluate its clinical relevance.

## 1. Introduction

Renal cell carcinoma (RCC) affects over 400,000 people globally each year [1]. Clear cell renal cell carcinoma (ccRCC) represents the most common and aggressive form of RCC, comprising almost 70%–75% of cases [2]. Despite advancements in diagnostic and therapeutic technologies, patients with ccRCC face a poor prognosis due to challenges such as metastatic spread and radiochemoresistance [3]. At the time of diagnosis, around 25%–30% of patients with RCC are found to have locally advanced or metastatic disease [4]. While targeted therapies, particularly tyrosine kinase inhibitors (TKIs) like sorafenib, have shown initial effectiveness, acquired drug resistance frequently limits their long-term efficacy [5]. Therefore, in order to improve therapy options and counteract the development and resistance observed in ccRCC, finding new biomarkers and therapeutic targets is of utmost importance.

KCNJ2, encodes the Kir2.1 protein, a key member of the inwardly rectifying potassium channel family [6], essential for potassium ion regulation and cellular excitability in diverse tissues such as neurons, skeletal muscle, cardiac myocytes, immune, and carcinoma cells [7]. In cancer, KCNJ2 has been implicated in various malignancies, exhibiting oncogenic effects by influencing cell proliferation and survival [1, 8, 9]. For instance, in medulloblastoma, KCNJ2 shows elevated expression specially in non-WNT/SHH subgroups, where it plays a key role in driving tumor cell invasion and metastasis, and this process is facilitated activation of the Notch2 signaling pathway [10]. In gastric cancer, KCNJ2 silencing reduces cell invasiveness, acting independently of potassium transport by its interaction with serine/threonine–protein kinase 38 [8]. Additionally, in osteosarcoma, KCNJ2 overexpression correlates with advanced disease and poor patient outcomes, as it enhances metastasis by forming a positive feedback loop with HIF-1*α* [11]. However, the roles of KCNJ2 in ccRCC remain largely unexplored. These examples highlight the urgent need to investigate KCNJ2's function in ccRCC to gain a better understanding of its potential influence on tumor progression and therapeutic strategies.

This study is aimed at exploring KCNJ2 expression and its clinical relevance in ccRCC by analyzing data from The Cancer Genome Atlas (TCGA) database. We then further explored the functional role of KCNJ2 and elucidated its underlying mechanism in vitro.

## 2. Methods

### 2.1. Data Source, Differential Expression, Clinicopathological Characteristics, and Coexpression Network Analysis of KCNJ2

This study utilized data from TCGA database (https://portal.gdc.cancer.gov/, accession number phs000178), including 614 cases with gene expression and clinical data (72 normal samples and 542 tumor samples). Data processing and organization were conducted using R software (v4.3.1). Differential expression analysis of KCNJ2 between ccRCC and normal tissues was performed using the TCGA-KIRC dataset [12] and the “limma” R package, with criteria of |logFC| < 1 and *p* value < 0.05.

Clinicopathological characteristics, including patient age, gender, metastatic status, cancer stages, and grade, were also extracted from the TCGA-KIRC cohort. Group differences were analyzed using the “limma” and “ggpubr” R packages. Survival outcomes, such as overall survival (OS) and progression-free survival (PFS), were analyzed using the “survival” and “survminer” packages to generate Kaplan–Meier (K-M) curves. Additionally, the “timeROC” R package was employed to evaluate the diagnostic value of KCNJ2 expression levels for ccRCC patient survival through receiver operating characteristic (ROC) curves.

A coexpression network was constructed using gene expression data from the TCGA-KIRC cohort. Genes coexpressed with KCNJ2 were identified using the “ggplot2,” “ggpubr,” and “ggExtra” R packages, focusing on those with correlation coefficients > 0.6 and *p* values < 0.001.

### 2.2. Tumor Microenvironment (TME), Cell Lines, and Cell Transfection

TME, where tumor cells reside, comprises a complex network of stromal cells (including fibroblasts, lymphocytes, macrophages, and endothelial cells), immune cells (such as T and B lymphocytes), and extracellular components (such as cytokines, growth factors, hormones, and the extracellular matrix (ECM)). These components encase the tumor cells and receive nourishment from the vascular system. The expression levels of KCNJ2 in relation to immune cell and stromal cell scores across different samples, using the R packages “reshape2,” “ggpubr,” and “limma,” determine if there were differences in KCNJ2 expression in stromal and immune cells. Human ccRCC cell lines 786-O and A498, acquired from the American Type Culture Collection (ATCC, United States), were cultured in RIPA-1640 medium (Gibco, United States) with 10% fetal bovine serum (FBS) (Gibco, United States) and 1% penicillin–streptomycin solution (Sigma, St. Louis, Missouri, United States). HK-2 human renal proximal tubular epithelial cell line was obtained from the Shanghai Institute for Biological Sciences Cell Resource Center and cultured in Dulbecco's Modified Eagle Medium/Nutrient Mixture F-12 (DMEM/F-12, Gibco, United States) supplemented with 10% FBS and 1% penicillin–streptomycin antibiotics. Additionally, the human RC cell lines ACHN and Caki-1 were also sourced from ATCC and cultured in DMEM with 10% FBS and 1% penicillin–streptomycin antibiotics. Cell lines were cultured at 37°C in a humidified atmosphere with 5% CO_2_. Two double-stranded siRNA oligonucleotides targeting KCNJ2 (KCNJ2-siRNA) were created and chemically produced in order to suppress KCNJ2 expression (Shanghai GenePharma Co). Cells were transfected with siRNA utilizing Lipofectamine RNAimax reagent (Thermo Fisher Scientific, United States). A pcDNA 3.1 vector-based plasmid (Beijing, Sino Biological) was developed and produced to overexpress KCNJ2. Cells were transfected with the KCNJ2 overexpression plasmid (OE) and a negative control plasmid (OE-NC) using Lipofectamine 3000 Transfection Reagent (Thermo Fisher Scientific, United State) following the manufacturer's instructions. The targeting sequences chosen were siRNA-NC 5⁣′-UUCUCCGAACGUGUCACGUTT-3⁣′; KCNJ2-siRNA 5⁣′-GUAAUGUUCAGUUCAUCAAUGTT-3⁣′. Cells were plated at a density of 1000 cells per well in 96-well plates. The cell proliferation rate was assessed using the CCK-8 assay (Dojindo, Japan). CCK-8 reagent was administrated to the 786-O and A498 cell lines after 0, 24, 48, and 72 h of incubation. The absorbance of the plates was measured at 450 nm following a 2-h culture period. For the cell proliferation assay, each condition was tested in three independent experiments, with triplicates for each experimental group in each run. The average values and standard deviations (SDs) from these replicates are reported. To ensure accuracy and reproducibility, ATP and glucose content were normalized to total protein concentration, which was measured using a bicinchoninic acid (BCA) assay for each sample. This normalization ensures that the reported values accurately reflect metabolic changes relative to cellular content. These details have been applied consistently across all relevant assays.

#### 2.2.1. Transfection Efficiency and Validation of KCNJ2 Knockdown/Overexpression

To investigate the functional role of KCNJ2 in ccRCC, KCNJ2 knockdown and overexpression were achieved in A498 cells using siRNA-mediated knockdown and plasmid-based overexpression, respectively. A498 cells were transfected with 100 nM of KCNJ2-specific siRNA (siKCNJ2) or 2 *μ*g of KCNJ2 overexpression plasmid (pcDNA3.1-KCNJ2) using Lipofectamine 3000 transfection reagent (Invitrogen), following the manufacturer's protocol. Control cells were transfected with nontargeting siRNA (siNC) or an empty vector plasmid (pcDNA3.1).

#### 2.2.2. Transfection Efficiency Validation

To confirm the efficiency of KCNJ2 knockdown and overexpression, both mRNA and protein levels were evaluated 48 h posttransfection. KCNJ2 mRNA expression was assessed by quantitative reverse transcription polymerase chain reaction (qRT-PCR), while KCNJ2 protein expression was analyzed using Western blotting.

### 2.3. qRT-PCR

The PrimeScript RT-PCR kit (Takara, China) was used to reverse transcribe the total RNA into cDNA after it had been extracted using the Trizol technique (Takara Bio, China). The cDNA was then amplified using SYBR qPCR Master Mix on a Bio-Rad Real-Time PCR system (United States), using GAPDH as the loading control. Gene primer sequences are illustrated below: GAPDH forward 5⁣′-GGAGCGAGATCCCTCCAAAAT-3⁣′, reverse 5⁣′-GGCTGTTGTCATACTTCTCATGG-3⁣′; KCNJ2 forward 5⁣′-CGGTGGATGCTGGTTATC-3⁣′, reverse 5⁣′-CTCAATGGAGAAGAGGAAGG-3⁣′; BAX forward 5⁣′-TGCTTCAGGGTTTCATCCAGG-3⁣′, reverse 5⁣′-TGAGACACTCGCTCAGCTTCT-3⁣′; BCL2 forward 5⁣′-CGACTTCGCCGAGATGTCCAGC-3⁣′, reverse 5⁣′-CGAACTCAAAGAAGGCCACAAT-3⁣′; E-cadherin forward 5⁣′-CCAAAGCCTCAGGTCATAAACA-3⁣′, reverse 5⁣′-TTGGGTCGTTGTACTGAATGGT-3⁣′; N-cadherin forward 5⁣′-TCCTGCTTATCCTTGTGCTGAT-3⁣′, reverse 5⁣′-CATAGTCCTGGTCTTCTTCTCC-3⁣′.

### 2.4. Comprehensive Analysis for Protein Expression, Cellular Behavior, and Metabolic Activity

Cells were lysed using RIPA lysis buffer (Beyotime, China), supplemented with protease inhibitors PMSF and a cocktail (MERCK, Germany). Proteins were equally loaded, separated using 10% SDS-PAGE, and transferred onto a PVDF membrane. Western blot analysis was performed using standard procedures. Antibodies used included anti-KCNJ2, anti-BAX, anti-BCL2, anti-E-cadherin, anti-N-cadherin, and anti-GAPDH, all sourced from Cell Signaling Technology (CST, United States). Cells were collected in ice-cold PBS following PBS washing. The cells were resuspended and incubated in a binding buffer with Annexin V and propidium iodide staining solution and incubated for 15 min in the dark, after three times PBS washes. The proportion of apoptotic cells was determined using flow cytometry (BD Biosciences, United States) following the manufacturer's instructions. Cells were seeded in six-well plates until reaching full confluence, after which a 200-*μ*L pipette was used to create a scratch and form a gap. The plates were incubated in a cell incubator for 48 h after switching to serum-free medium. Scratch images were captured with an inverted microscope. Boyden chambers (Millipore, Germany) were inserted onto 24-well plates with pores as small as 8 *μ*m. The procedure involved seeding 8 × 10^6^ cells in the upper chamber using Matrigel in 200 *μ*L of culture media without serum. After a predetermined amount of time, the chambers were fixed, cleaned with PBS, and dyed using 0.1% crystal violet diluted in 4% paraformaldehyde. An inverted light microscope set to ×100 magnification was then used to record and count visible cells in five randomly selected fields. The intracellular ATP content was measured using an ATP assessment kit (Elabscience, China). Firstly, cells were collected and treated using 0.3 mL of reagent one for every 2 × 10^6^ cells in a 2-mL EP tube. The tube was tightly capped, mixed thoroughly, and then placed in boiling water for a 10-min water bath. After cooling under running water, the samples were centrifuged at 4°C for 5 min at 12,000 *g*, and the supernatant was collected for analysis. A plate's corresponding wells were filled with 100 *μ*L of the enzyme working solution for the experiment, which was then let to stand for 5 min. The standard wells were then rapidly mixed with 100 *μ*L of standards at different concentrations. The sample supernatant (100 *μ*L) was added to the measurement wells and mixed similarly. A chemiluminescence detection device was used to measure the fluorescence values for every well. To measure the cellular glucose level, cells were washed twice with PBS and lysed using 200 *μ*L of lysis buffer per well in a six-well plate. Following centrifugation at 12,000 *g* for 5 min, 5 *μ*L of the supernatant was placed into a PCR tube. Depending on the glucose concentration, 20 *μ*L or smaller volumes could be used. To each tube, 185 *μ*L of Glucose Assay Reagent (Beyotime, China) was added, adjusted to a final volume of 190 *μ*L, followed by vortex mixing and brief centrifugation. The samples were subjected to a temperature of 95°C for 8 min and then cooled to 4°C, before transferring 180 *μ*L to a new 96-well plate. Absorbance at 630 nm was recorded within 30 min, and glucose concentrations were calculated via a standard curve. Cells were cultured at a density of 10^4^ cells per well in a 96-well plate. After a 48-h treatment with the specified conditions, cells were harvested, and cell lysis was transferred to a clear 96-well plate. Then, 50 *μ*L of LDH reaction working buffer (Elabscience, China) was added to each well and allowed to incubate at 25°C for 30 min. Absorbance at 450 nm was recorded using an ELISA reader (Thermo, United States).

### 2.5. Statistical Analysis

All statistical analyses and visualizations in this study were performed using Prism version 8.0 and R software version 4.3.1. The Wilcoxon test was employed to compare two groups, while the Kruskal–Wallis test was performed to compare multiple groups. Continuous variables were presented as mean ± SD, and categorical variables were expressed as counts and percentages. Student's *t*-test assessed group differences, with statistical significance set at *p* value < 0.05. To assess the correlation between KCNJ2 expression and clinical characteristics, we used the Kruskal–Wallis test for nonparametric comparisons across multiple subgroups (age, gender, tumor grade, metastasis status, and cancer stages). For pairwise comparisons of tumor sizes and stages, the Mann–Whitney *U* test was applied. All tests were two-tailed, with significance set at *p* < 0.05. Kaplan–Meier survival curves were constructed to evaluate OS and PFS based on the median KCNJ2 expression levels. Log-rank tests were used to compare survival between high and low KCNJ2 expression groups. Hazard ratios (HRs) and 95% confidence intervals (CIs) were calculated using Cox proportional hazard regression models to estimate the prognostic value of KCNJ2 for OS and PFS. ROC curve analysis was employed to assess the predictive accuracy of KCNJ2 for survival outcomes (1-, 3-, and 5-year OS and PFS). The area under the ROC curve (AUC) was calculated to quantify the performance of KCNJ2 as a prognostic biomarker. Pearson correlation analysis was performed to investigate the correlation between KCNJ2 expression and other gene expressions. Genes with significant positive correlations (*p* < 0.001) were further analyzed, and the results were presented in a coexpression network. The relationship between KCNJ2 expression and immune infiltration was assessed using the ESTIMATE algorithm to calculate immune, stromal, and estimate scores. Comparison between high and low KCNJ2 expression groups was performed using the Wilcoxon test. The proportions of immune cells were determined using the CIBERSORT algorithm, and the differences in immune cell infiltration between KCNJ2 high- and low-expression groups were compared using the Wilcoxon rank-sum test. Statistical differences in cell proliferation (CCK-8 assay), migration, and invasion (transwell assays) and apoptosis (flow cytometry) between KCNJ2 overexpressed and knockdown groups were analyzed using one-way ANOVA followed by post hoc Tukey's test. The expression levels of genes associated with apoptosis, migration, and invasion in ccRCC cells were evaluated using qRT-PCR and Western blot. Data were normalized to the internal control gene (GAPDH), and statistical significance was assessed by one-way ANOVA or *t*-tests as appropriate.

## 3. Results

### 3.1. KCNJ2 Expression Was Elevated in ccRCC

**Figure 1:**
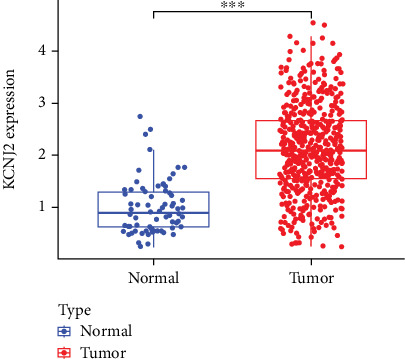
High expression of KCNJ2 in ccRCC. ⁣^∗∗∗^*p* < 0.001 versus normal kidney tissues.

We analyzed TCGA database to examine KCNJ2 expression differences between ccRCC and normal kidney tissues.  Figure 1 demonstrates a significant upregulation of KCNJ2 expression in ccRCC tissues (*p* < 0.001).

### 3.2. KCNJ2 Expression Across Subgroups With Varying Clinical Characteristics

**Figure 2:**
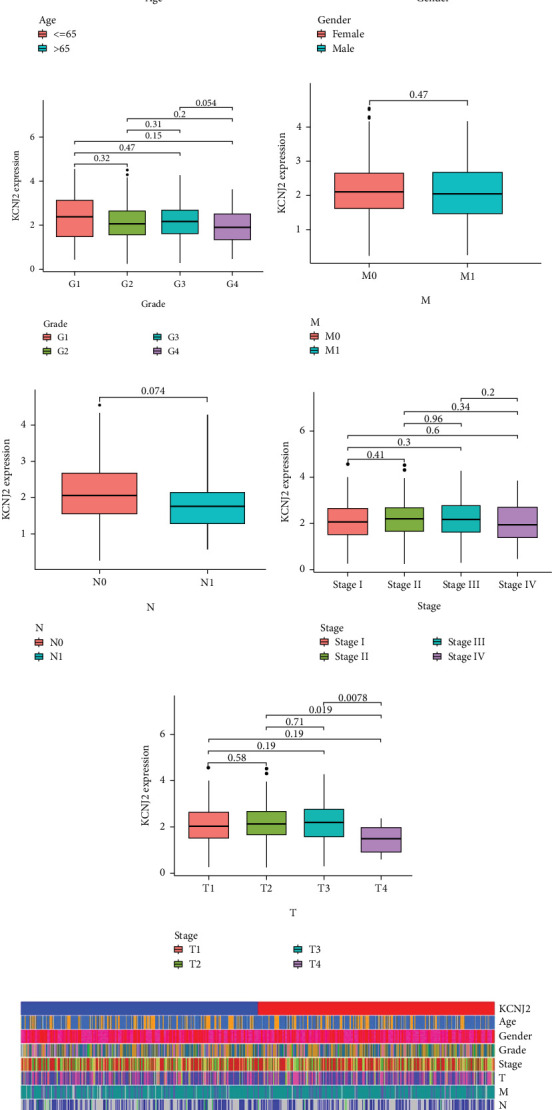
KCNJ2 expression across subgroups with different clinical characteristics. Box plots depict the relative mRNA expression of KCNJ2 across various ccRCC patient groups: (a) age, (b) gender, (c) tumor grade, (d) distant metastasis status, (e) nodal metastasis status, (f) cancer stages, (g) primary tumor size and extent, and (h) comparison of clinical characteristics between KCNJ2 high and low expression subgroups.

As illustrated in Figures 2a, 2b, 2c, 2d, 2e, and 2f, KCNJ2 expression did not show a correlation with age, gender, tumor grade, distant metastasis, nodal metastasis, or cancer stage (all *p* > 0.05). However, significant differences in KCNJ2 mRNA levels were observed when comparing the primary tumor size and extent, specifically between T1 and T4 stages, T2 and T4 stages, and T3 and T4 stages (all *p* < 0.05) (Figure 2g,h). According to these results, negative clinical–pathological characteristics are linked to KCNJ2 expression, and more advanced malignant progression of ccRCC is indicated by greater KCNJ2 levels.

### 3.3. Prognostic Significance of KCNJ2 in ccRCC

**Figure 3:**
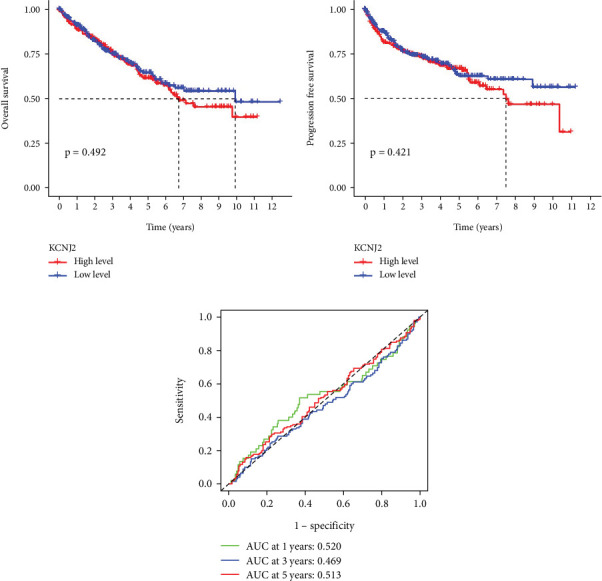
Prognostic significance of KCNJ2 in ccRCC. (a) Kaplan–Meier survival curve analysis of OS. (b) Kaplan–Meier survival curve analysis of PFS. (c) ROC curve analysis of the survival rate.

The association between KCNJ2 level and survival outcomes in ccRCC patients was analyzed using Kaplan–Meier survival curves and ROC curves from the TCGA-KIRC cohort. ccRCC patients were categorized into two groups according to median KCNJ2 mRNA levels, as illustrated in Figure 3a,b. KCNJ2 expression showed no discernible variations in the OS and PFS. As illustrated in Figure 3c, the survival projections at 1, 3, and 5 years based on KCNJ2 levels were generally modest. These findings suggest that KCNJ2 may not serve as an effective and predictive biomarker for ccRCC.

### 3.4. Constructing Gene Coexpression Networks

We examined the coexpressed of genes with KCNJ2 by analyzing the expression levels of all genes across the samples.  Table 1 reveals 38 genes positively coexpressed with KCNJ2 (*p* < 0.001).

### 3.5. Analysis of TME and the Correlation Between KCNJ2 Expression, Immune Marker Expression, and Immune Checkpoints

**Figure 4:**
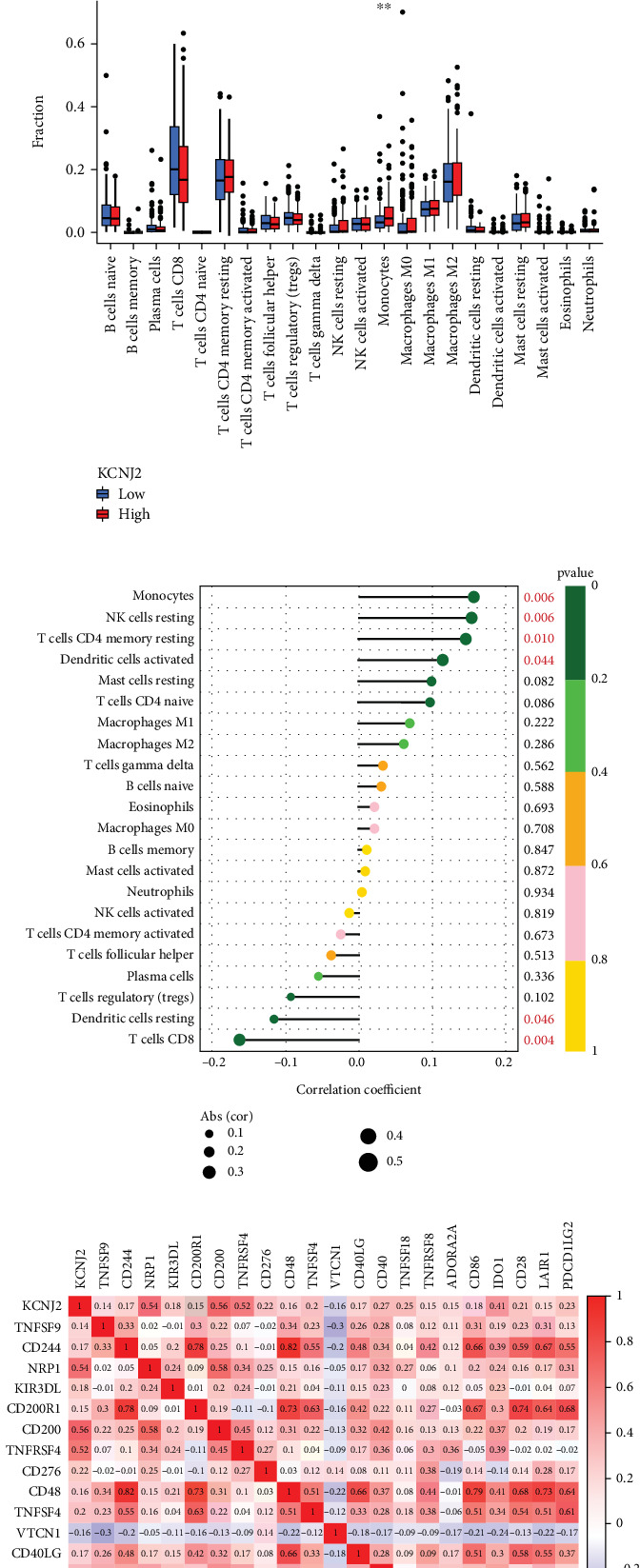
Associations between KCNJ2 expression with immune infiltration and immune checkpoints in ccRCC. (a–c) Analysis of the relationship between immune infiltration level and KCNJ2 expression in ccRCC patients. (d) Heatmap illustrating the correlation between KCNJ2 expression and immune checkpoints utilizing the TCGA-KIRC dataset. ⁣^∗^*p* < 0.05, ⁣^∗∗^*p* < 0.01, and ⁣^∗∗∗^*p* < 0.001.

Figure 4a illustrates the evaluation of the estimate, immune, and stromal scores in subgroups with high and low KCNJ2 expression, determined by the median expression level of KCNJ2. Our study indicated a significant positive correlation between KCNJ2 expression and the estimate, immune, and stromal scores (all *p* < 0.05). We also evaluated the infiltration proportions of 22 immune cell types in ccRCC by comparing KCNJ2 high- and low-expression groups, using the median KCNJ2 expression level from the TCGA-KIRC cohort as a cutoff.  Figure 4b demonstrates that patients with high KCNJ2 expression had significantly elevated levels of monocyte infiltration (*p* < 0.05).  Figure 4c demonstrates that KCNJ2 expression positively correlated with monocytes, resting NK cells, resting memory CD4 cells, and activated dendritic cells (DCs) (all *p* < 0.05) and negatively correlated with resting DCs and CD8 T cells (all *p* < 0.05). These findings indicate a link between KCNJ2 and immune cell infiltration as well as TME.

The correlation analysis between KCNJ2 expression and various immunoinhibitors and immunostimulators is presented as heatmaps in Figure 4d. The results indicate a strong relationship between KCNJ2 and several immune checkpoints, including Neuropilin 1 (NRP1), CD200 molecule (CD200), and TNF receptor superfamily member 4 (TNFRSF4).

### 3.6. Impacts of KCNJ2 on Cell Growth, Apoptosis, Migration, and Invasiveness in ccRCC

**Figure 5:**
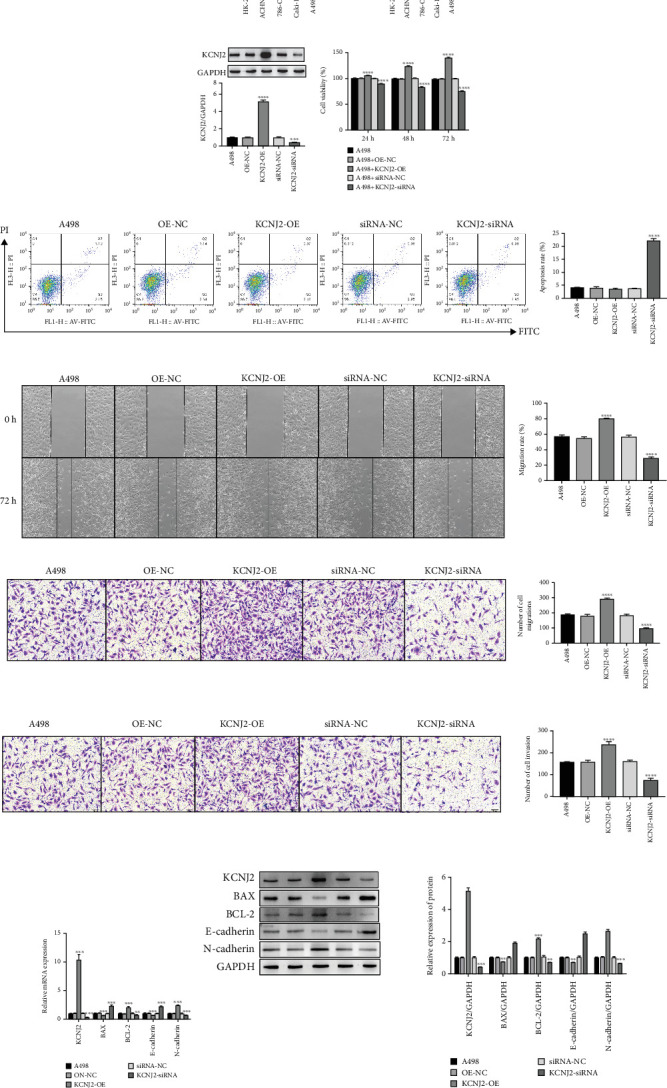
Impacts of KCNJ2 on cell growths, apoptosis, migration, and invasiveness in ccRCC. (a, b) qRT-PCR and Western blot analysis of KCNJ2 mRNA and protein levels in ccRCC cell lines and the human renal tubular epithelial cell line. (c) Western blot analysis of the efficiency of KCNJ2 overexpression or knockdown. (d) Cell viability of A498 cells was measured by CCK-8 assay. (e) Cell apoptosis rate of A498 cells was measured by flow cytometry assay. (f) Wound healing in A498 cells. (g, h) Cell migration and invasion abilities were detected by transwell assays. (i, j) The mRNA and protein changes of apoptosis, migration, and invasion-related genes were detected by qRT-PCR and Western blot assay. ⁣^∗∗^*p* < 0.01 and ⁣^∗∗∗^*p* < 0.001.

Our findings indicated that all ccRCC cell lines exhibited significantly higher KCNJ2 mRNA and protein levels compared to HK-2 cells (all *p* < 0.001, Figure 5a,b). To investigate KCNJ2's role in ccRCC, we conducted gain- and loss-of-function assays by suppressing and overexpressing KCNJ2 in A498 cells. The efficiency of KCNJ2 knockdown and overexpression was verified via Western blot analysis (Figure 5c). The CCK-8 assay showed that KCNJ2 knockdown in A498 cells leads to a significant, time-dependent reduction in cell proliferation (all *p* < 0.001, Figure 5d). In contrast, A498 cells with KCNJ2 overexpression exhibited a significant increase in proliferative capacity (all *p* < 0.001, Figure 5d).

Flow cytometry assay was used to detect whether KCNJ2 influences the apoptosis of ccRCC cells.  Figure 5e illustrates that suppression of KCNJ2 expression obviously increased the apoptosis rate of A498 cells (*p* < 0.001). We conducted wound healing and transwell assays to investigate the KCNJ2's role in ccRCC cell migration and invasion. The findings demonstrated KCNJ2 knockdown markedly hindered A498 cells' wound healing capacity (*p* < 0.001), while its overexpression enhanced this ability (*p* < 0.001, Figure 5g). Furthermore, silencing KCNJ2 expression inhibited both the migration and invasion capacities of A498 cells (all *p* < 0.001), while KCNJ2 overexpression enhanced these abilities (all *p* < 0.001, Figure 5h).

We employed qRT-PCR and Western blot assays to assess mRNA and protein alterations in genes associated with apoptosis, migration, and invasion. Our findings indicated that overexpression of KCNJ2 decreased the levels of BAX and E-cadherin while increasing the levels of BCL-2 and N-cadherin (all *p* < 0.01, Figure 5i,j). Conversely, inhibition of KCNJ2 produced the opposite effects (all *p* < 0.01, Figure 5i,j).

### 3.7. Effects of KCNJ2 on Glucose Metabolism in ccRCC Cells

**Figure 6:**
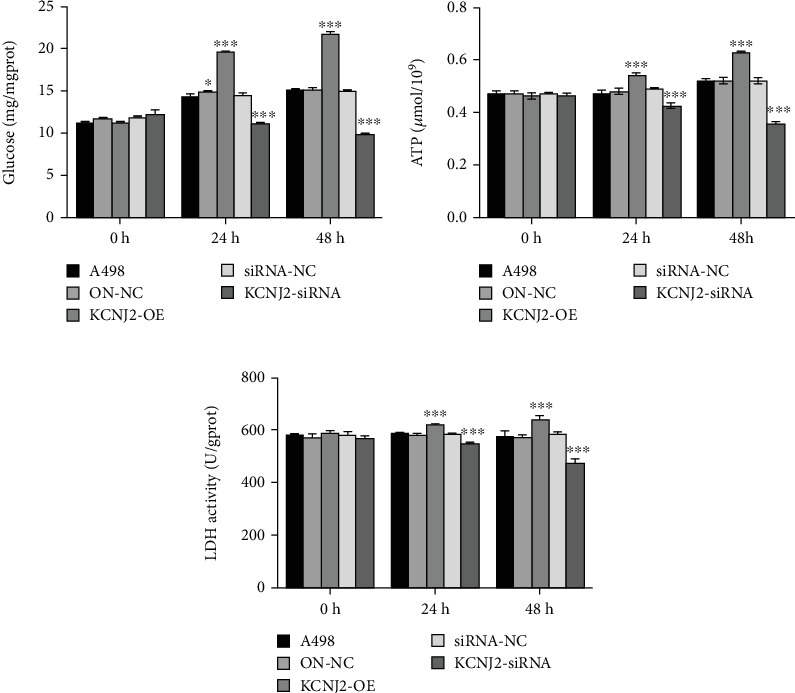
Impacts of KCNJ2 on glucose metabolism in ccRCC cells. (a–c) Cellular glucose levels, ATP levels, and LDH activity of A498 cells. ⁣^∗^*p* < 0.05 and ⁣^∗∗∗^*p* < 0.001.

Coexpression network analysis revealed a correlation between KCNJ2 expression and several energy metabolism genes, such as Rap guanine nucleotide exchange factor 5 (RAPGEF5) [13], signal-induced proliferation-associated 1 like 2 (SIPA1L2), and phosphoglucomutase 2 like 1 (PGM2L1), which are linked to GTP and glucose metabolism [13–15]. We examined KCNJ2's impact on glucose metabolism in A498 cells by assessing intracellular glucose production, LDH activity, and ATP generation. As shown in Figures 6a, 6b, and 6c, our results demonstrated that overexpression of KCNJ2 significantly increased glucose production, intracellular ATP levels, and LDH activity in A498 cells (all *p* < 0.001). In contrast, inhibiting KCNJ2 resulted in significantly different outcomes (all *p* < 0.001).

## 4. Discussion

This study examined KCNJ2's role in ccRCC, revealing that higher KCNJ2 expression is linked to advanced malignancy, immune cell infiltration, and unique features of the TME. Furthermore, our results indicate that KCNJ2 may influence key pathways related to proliferation, migration, invasion, apoptosis, and glucose metabolism in ccRCC cells, thereby contributing to the aggressive phenotype of this cancer.

KCNJ2, part of the classical inward rectifying potassium channel subfamily, facilitates inward rectifying potassium currents in diverse cell types such as neurons, skeletal muscle cells, cardiac myocytes, immune cells, and cancer cells [16, 17]. Prior research has demonstrated that KCNJ2 is capable of advancing the progression of multiple cancer types, regardless of the underlying molecular mechanisms associated with its inward rectifying potassium current [8, 10, 11]. Recent studies have highlighted the role of KCNJ2 in various cancers beyond the primary focus of this study. For example, KCNJ2 has been implicated in promoting epithelial–mesenchymal transition and metastasis in osteosarcoma [11]. In thyroid carcinoma, KCNJ2 inhibits proliferation, migration, and EMT progression of papillary cells by upregulating GNG2 expression [18]. These findings suggest that KCNJ2 may have a broader oncogenic role across multiple cancer types. Incorporating this broader context strengthens the rationale for exploring KCNJ2 as a potential biomarker or therapeutic target in oncology. For instance, Liu et al. identified an upregulation of KCNJ2 in small-cell lung cancer tissues, which facilitated multidrug resistance via Ras/MAPK signaling pathway activation [9]. Similarly, Chen et al. revealed that the inhibition of KCNJ2 could impede the epithelial–mesenchymal transition in papillary thyroid carcinoma cells by upregulating G protein subunit gamma 2 expression [18]. Additionally, Ji et al. reported that KCNJ2 could interact with and activate serine/threonine kinase 38, promoting gastric cancer cell invasion [8]. In our research, we observed that KCNJ2 levels were significantly increased in advanced ccRCC tissues. While we did not find a notable prognostic value for KCNJ2 in this context, our results indicate that it was associated with immune cell infiltration, characteristics of TME, and specific immune checkpoints. The results indicate that KCNJ2 potentially acts as an oncogene in ccRCC initiation and progression.

Despite the lack of significant prognostic value for KCNJ2 in the Kaplan–Meier survival analysis, its functional relevance should not be overlooked. KCNJ2's associations with critical signaling pathways, including those involved in tumor progression and immune regulation, suggest it may play an important role in ccRCC biology. For instance, its correlation with immune checkpoint markers implies a possible involvement in immune evasion mechanisms, as discussed earlier. This functional importance highlights the need for further experimental studies to investigate KCNJ2's role in tumor cell behavior and the TME. While it may not independently predict patient outcomes, KCNJ2 could act as a contributing factor in the complex network of molecular events driving ccRCC progression. Such insights could inform future therapeutic strategies targeting ion channels or immune–modulatory pathways.

To investigate the molecular mechanisms underlying the effects of KCNJ2, we conducted a coexpression network analysis. The analysis identified an association between KCNJ2 and genes related to energy metabolism, including RAPGEF5, SIPA1L, and PGM2L1, which were linked to GTP and glucose metabolism [13–15]. Furthermore, in vitro studies with A498 cells demonstrated that elevated KCNJ2 expression led to increased glucose production, LDH activity, and ATP generation, thereby promoting the aggressive characteristics of ccRCC cells.

The TME, consisting of stromal cells, fibroblasts, mesenchymal stem cells, adipocytes, endothelial cells, and diverse immune cells, is critical in influencing tumor behavior [19]. The reprogramming of this microenvironment significantly influences tumor response to therapies such as chemotherapy, radiotherapy, targeted treatments, and immune checkpoint inhibitors, all of which are modulated by metabolic processes including glycolysis, glutaminolysis, and fatty acid metabolism [20]. As research in this area progresses, the metabolic influences on the TME are increasingly recognized as pivotal [21]. Given that kidney cancer is characterized by disrupted cell cycle regulation and metabolic reprogramming, it can be classified as a metabolic disease [22]. ccRCC often shows modifications in glucose and fatty acid metabolism, along with alterations in the tricarboxylic acid cycle [22]. Additionally, our findings indicated that KCNJ2 mRNA levels were correlated with the monocyte fraction and several immune checkpoints, including NRP1, CD200, and TNFRSF4. This suggests that KCNJ2's role in immune modulation may also impact the progression of ccRCC.

KCNJ2's role in enhancing glucose metabolism may be linked to its regulation of cellular ion homeostasis, which is known to influence metabolic processes. One plausible mechanism involves the hypoxia-inducible factor-1*α* (HIF-1*α*) signaling pathway, a critical regulator of metabolic reprogramming in tumors. HIF-1*α* is upregulated in hypoxic environments and promotes glycolysis by activating glucose transporters and glycolytic enzymes. While our study does not directly evaluate the interplay between KCNJ2 and HIF-1*α*, prior evidence suggests that ion channel activity can modulate HIF-1*α* stability and activity. Future studies could investigate whether KCNJ2 directly or indirectly influences glucose metabolism through this pathway, potentially contributing to the Warburg effect in ccRCC. The observed correlation between KCNJ2 expression and immune checkpoint markers suggests a possible role in modulating the tumor immune microenvironment. KCNJ2 may influence immune cell infiltration or function via alterations in the ionic composition of the TME, which can affect T cell activation, differentiation, and effector functions. For instance, potassium ion flux, regulated by channels such as KCNJ2, has been shown to impact T cell metabolism and cytokine production. Additionally, KCNJ2-mediated changes in metabolic activity, such as enhanced glycolysis, could create a competitive metabolic landscape, potentially limiting nutrient availability for immune cells. These interactions merit further exploration to delineate the precise mechanisms by which KCNJ2 influences immune evasion and tumor progression.

Our findings not only align with existing research on the role of KCNJ2 in cancer but also offer new insights into its specific functions in ccRCC. KCNJ2 expression was markedly higher in ccRCC tissues than in normal kidney tissues, suggesting a distinct regulatory mechanism that warrants further investigation. Additionally, the association of KCNJ2 with glucose metabolism and immune regulation within the context of ccRCC underscores its intricate role in cancer biology. This specificity highlights the multifaceted functions of KCNJ2 and underscores the necessity for targeted investigations to clarify how different ion channels may exert varied effects across distinct cancer types.

### 4.1. Limitations of the Study

While this study provides valuable insights into the role of KCNJ2 in ccRCC, several limitations should be considered when interpreting the findings. Firstly, the study primarily relied on data from the TCGA-KIRC cohort, which may limit the generalizability of the results to broader patient populations. The relatively small sample size in some of the subgroups may also reduce the statistical power of certain analyses, particularly when examining the correlation between KCNJ2 expression and clinical outcomes such as OS and PFS. Additionally, while we have identified several significant correlations between KCNJ2 expression and clinical characteristics, causality cannot be established in this observational study. Future studies incorporating prospective cohorts or randomized controlled trials (RCTs) would help confirm these findings and their relevance to clinical practice. Finally, the study lacks in vivo validation to assess the functional role of KCNJ2 in a more physiologically relevant context, and clinical samples were not included, which limits the direct translational implications of our findings.

### 4.2. Suggestions for Future Research

Future research could address these limitations by employing larger, multicenter cohorts to enhance the generalizability of the findings. Additionally, functional studies examining KCNJ2 at both the mRNA and protein levels in ccRCC tissue samples are crucial for confirming its role in tumor progression and prognosis. We recommend the use of animal models and patient-derived xenografts (PDXs) to evaluate the functional impact of KCNJ2 modulation on ccRCC progression, metastasis, and response to therapy.

In terms of clinical relevance, future studies should investigate the potential of KCNJ2 as a prognostic biomarker for ccRCC by assessing its performance in predicting patient outcomes in larger, independent cohorts. It would be beneficial to integrate KCNJ2 expression with other established biomarkers and clinical factors to create more accurate predictive models for patient stratification and treatment decision-making.

Furthermore, understanding the mechanistic relationship between KCNJ2 and immune cell infiltration in the TME offers exciting opportunities. Investigating how KCNJ2 influences immune evasion mechanisms or response to immunotherapy could provide novel therapeutic strategies for ccRCC patients. Exploring the role of KCNJ2 in metabolic reprogramming, especially in the context of glucose metabolism, could also uncover new targets for treatment, potentially improving therapeutic outcomes for patients with advanced ccRCC.

Finally, it would be of interest to explore the potential of combining KCNJ2 expression with other genetic and molecular markers to predict patient responses to targeted therapies and immunotherapies, ultimately guiding personalized treatment approaches for ccRCC patients.

## 5. Conclusion

Our findings identified KCNJ2 as a key contributor to of ccRCC progression by regulating glucose metabolism and immune responses, highlighting its potential as a therapeutic target. Future studies should investigate the intricate mechanisms underlying KCNJ2's role in ccRCC and evaluate its potential for clinical application. Deepening our understanding of ion channel–related genes in ccRCC could lead to the development of effective therapeutic strategies, ultimately enhancing patient outcomes.

## Figures and Tables

**Table 1:** Coexpression networks with KCNJ2.

**Gene symbol**	**Correlation coefficient**	**p** ** value**	**Gene symbol**	**Correlation coefficient**	**p** ** value**
DLL4	0.7137688	1.45E − 85	LAMA4	0.6029844	5.79E − 55
MYCT1	0.6167598	4.13E − 58	ANGPTL2	0.6293355	4.01E − 61
P2RY1	0.7074633	1.94E − 83	GJA1	0.6505877	1.53E − 66
PECAM1	0.6223071	2.01E − 59	ADGRL4	0.6576115	1.98E − 68
NRARP	0.6633273	5.28E − 70	RHOJ	0.6708118	4.06E − 72
FOLH1	0.6009809	1.61E − 54	CDH5	0.6278005	9.51E − 61
MEF2C	0.6517781	7.39E − 67	ERG	0.6080402	4.22E − 56
GNG2	0.6095432	1.92E − 56	ANGPT2	0.7366574	8.53E − 94
KCNE3	0.6943735	3.37E − 79	ADAMTS5	0.6208457	4.49E − 59
BCL6B	0.6260119	2.59E − 60	THSD1	0.6218734	2.55E − 59
ACKR3	0.6769974	6.51E − 74	PCDH17	0.7031193	5.26E − 82
EFNB2	0.6048191	2.25E − 55	CD93	0.6359466	9.19E − 63
NR5A2	0.6740672	4.67E − 73	ESM1	0.6054187	1.65E − 55
FLI1	0.6264243	2.05E − 60	TNFAIP8L1	0.6086219	3.12E − 56
FLT1	0.6261879	2.34E − 60	AFAP1L1	0.6006424	1.92E − 54
RAPGEF5	0.6329623	5.11E − 62	TCF4	0.6088050	2.83E − 56
ITGA1	0.6404515	6.65E − 64	RASGRP3	0.6040842	3.29E − 55
DIPK2B	0.6365583	6.45E − 63	SIPA1L2	0.6205533	5.26E − 59
PGM2L1	0.6500427	2.13E − 66	MCAM	0.6372829	4.23E − 63

## Data Availability

The research data supporting this study are openly available and adhere to the “FAIR” principles (findable, accessible, interoperable, and reusable). Raw data, processed data, software, algorithms, protocols, methods, and materials are available through appropriate channels. To access the data, please contact the corresponding authors, who can provide the necessary information and facilitate data retrieval.
